# Plant disease resistance outputs regulated by AP2/ERF transcription factor family

**DOI:** 10.1007/s44154-023-00140-y

**Published:** 2024-01-02

**Authors:** Ning Ma, Ping Sun, Zhao-Yang Li, Fu-Jun Zhang, Xiao-Fei Wang, Chun-Xiang You, Chun-Ling Zhang, Zhenlu Zhang

**Affiliations:** 1 College of Horticulture Science and Engineering, Apple Technology Innovation Center of Shandong Province, National Key Laboratory of Wheat Improvement, Shandong Agricultural University, Tai’an, 271000 Shandong China; 2 Department of Horticulture, College of Agriculture, Shihezi University, Shihezi, 832003 Xinjiang China; 3 College of Agricultural Science and Technology, Shandong Agriculture and Engineering University, Jinan, 250100 Shandong China

**Keywords:** AP2/ERFs, Plant defense responses, Defense phytohormones, Secondary metabolites, Physical barriers, MAPK cascades

## Abstract

Plants have evolved a complex and elaborate signaling network to respond appropriately to the pathogen invasion by regulating expression of defensive genes through certain transcription factors. The APETALA2/ethylene response factor (AP2/ERF) family members have been determined as key regulators in growth, development, and stress responses in plants. Moreover, a growing body of evidence has demonstrated the critical roles of AP2/ERFs in plant disease resistance. In this review, we describe recent advances for the function of AP2/ERFs in defense responses against microbial pathogens. We summarize that AP2/ERFs are involved in plant disease resistance by acting downstream of mitogen activated protein kinase (MAPK) cascades, and regulating expression of genes associated with hormonal signaling pathways, biosynthesis of secondary metabolites, and formation of physical barriers in an MAPK-dependent or -independent manner. The present review provides a multidimensional perspective on the functions of AP2/ERFs in plant disease resistance, which will facilitate the understanding and future investigation on the roles of AP2/ERFs in plant immunity.

## Introduction

Plants have developed elaborate and complex response mechanisms to cope with challenges of various pathogens (e.g. fungi, bacteria, and viruses). Pathogen-associated molecular pattern (PAMP)-triggered immunity (PTI) and effector-triggered immunity (ETI) are the two layers of immune systems that play critical roles in defense responses (Dangl, et al. 2013; Ngou, et al. 2022). PAMPs are perceived and recognized by pattern recognition receptors (PRRs) localized at the cell surface which initiate PTI, the first layer of plant immune system (Bernoux, et al. 2022; Yuan, et al. 2023). To survive and colonize host plants, pathogens often secrete virulence effectors into host cells to suppress PTI. Plants counteract the dampened PTI using resistance (R) genes which are often intracellular proteins with nucleotide-binding domain leucine-rich repeat (NLR) domains that directly or indirectly detect pathogen effectors and activate ETI (Jones, et al. 2016; Ngou, et al. 2022) (Table 1).

**Table 1:** A summary of the type of AP2/ERF family according to their source, domain type and associated defense responses

Species	AP2/ERF factor name	Subfamily	Domain type	Associated defense responses	Reference
*Oryza sativa*	OsEREBP1	ERF	AP2/EREB	MAPK	Cheong, et al. 2003
*Arabidopsis thaliana*	AtERF6	ERF	AP2/EREB	MAPK	Meng, et al. 2013
*Arabidopsis thaliana*	AtERF72	ERF	AP2/EREB	MAPK	Li, et al. 2022a, b
*Glycine max*	GmERF113	ERF	AP2/EREB	MAPK	Gao, et al. 2022
*Malus doemstica*	MdERF11	ERF	AP2/EREB	SA	Wang, et al. 2020
*Capsicum annuum L*	RAV1	RAV	AP2/EREB B3	SA	Sohn, et al. 2006
*Oryza sativa*	OsERF3	ERF	AP2/EREB	SA/JA/ET	Lu, et al. 2011
*Solanum lycopersicum*	SlERF2	ERF	AP2/EREB	SA/JA	Yang, et al. 2021a, b
*Vitis quinquangularis*	VqERF114	ERF	AP2/EREB	Secondary metabolites	Wang and Wang 2019
*Nicotiana tabcum*	NtERF1/32/121	ERF	AP2/EREB	Secondary metabolites	Sears, et al. 2014
*Gossypium barbadense*	GbERF1-like	ERF	AP2/EREB	Physical barriers	Guo, et al. 2016
*Arabidopsis thaliana*	AtERF114	ERF	AP2/EREB	Physical barriers	Li, et al. 2022a, b
*Malus doemstica*	MdERF114	ERF	AP2/EREB	Physical barriers	Liu, et al. 2023
*Solanum lycopersicum*	SlSHN3	ERF	AP2/EREB	Physical barriers	Buxdorf, et al. 2014

These two layers of immune system are commonly associated with various plant immune responses, such as accumulation of salicylic acid (SA), reactive oxygen species (ROS) production, and the expression of various defense-related genes (Greenberg and Yao 2004; van der Hoorn and Kamoun 2008). Generally, in the early stage of PTI, cellular responses include the activation of mitogen-activated protein kinase (MAPK), cytoplasmic Ca^2+^ influx, apoplastic alkalinization, stomata closure, membrane depolarization, production of ROS, and genome-wide transcriptional reprogramming (Bigeard, et al. 2015; Zhang, et al. 2023a, b, c). While in the late stage of PTI, cellular responses include biosynthesis of secondary metabolites and defense hormones SA and ethylene (ET), and deposition of callose and lignin in plant cell walls (Clay, et al. 2009; Zhang, et al. 2023a, b, c).

Transcription factors (TFs) are proteins that bind specifically to *cis*-regulatory elements in the promoter regions of genes, resulting in activation or suppression of gene expression (Pireyre and Burow 2015; Sohn, et al. 2006; Xing, et al. 2017). TF family APETALA2/ethylene response factors (AP2/ERFs) is a conserved superfamily widely involved in plant growth and development, including light acclimation, leaf senescence, flower pedicel abscission, and fruit ripening (Dey and Corina Vlot 2015; Koyama, et al. 2013; Nakano, et al. 2014; Vogel, et al. 2014; Zhai, et al. 2022). Moreover, AP2/ERFs also play key roles in regulating plant responses to various biotic and abiotic stresses, including salinity, drought, temperature, and multiple pathogens (e.g. viruses, fungi, and bacteria) (Qi, et al. 2023; Xie, et al. 2019). AP2/ERF superfamily is traditionally considered to specifically exist in plants, until its orthologs have been identified in genomes of microbes, such as the cyanobacterium *Trichodesmium erythraeum*, the virus *Enterobacteria phage Rb49* (Magnani, et al. 2004; Wessler 2005). To date, a huge number of genes encoding AP2/ERF proteins have been identified in multiple plant species, including *Solanum lycopersicum* (146 genes) (Pirrello, et al. 2012), *Malus domestica* (259 genes) (Girardi, et al. 2013), sweet orange (*Citrus sinensis*, 108 genes) (Ito, et al. 2014), and others. Different types of duplication events, such as tandem and segmental duplication, or whole-genome duplication may have contributed to AP2/ERFs expansion across plant kingdom (Zhang, et al. 2021a, b, c; Zhuang, et al. 2009). The duplication and expansion enable functional differentiation of AP2/ERF family members to regulate diverse cellular processes, one of the key functions being defense responses (Feng, et al. 2020; Gao, et al. 2020).

A rising body of evidence shows that AP2/ERFs are closely involved in defense responses against various pathogens in plants (Reboledo, et al. 2022; Zang, et al. 2021). For example, overexpressing *NtERF5* increases expression levels of pathogenesis-related (PR) genes and confers resistance to tobacco mosaic virus in tobacco (*Nicotiana tobacum*), while overexpressing *Pti4/5/6* confer increased resistance to *Erysiphe orontii*, and *Pseudomonas syringae* pv *tomato* in Arabidopsis (Fischer and Dröge-Laser 2004; Gu, et al. 2002). Thus, this review will focus on the roles of AP2/ERF family in plant immune responses, and summarize AP2/ERFs as direct targets of MAPK signaling and how they regulate genes associated with defense hormonal signaling pathways, synthesis of antimicrobial secondary metabolites and physical barriers.

## Features, classification, and binding specificity of AP2/ERF family

Member of AP2/ERF family encode a conserved APETALA2 (AP2)/Ethylene Responsive Element Binding Factor (EREB) domain, which contains 60–70 amino acid residues and is responsible for recognizing and binding to *cis*-regulatory elements in target genes (Nakano, et al. 2006; Okamuro, et al. 1997; Xie, et al. 2022). More specifically, arginine and tryptophan residues within the β-sheet of the AP2/EREB domain are pivotal for DNA binding (Aiese Cigliano, et al. 2013). In plants, the first AP2/EREB domain-containing protein was identified in model plant *Arabidopsis thaliana* (Jofuku, et al. 1994).

**Fig. 1:**
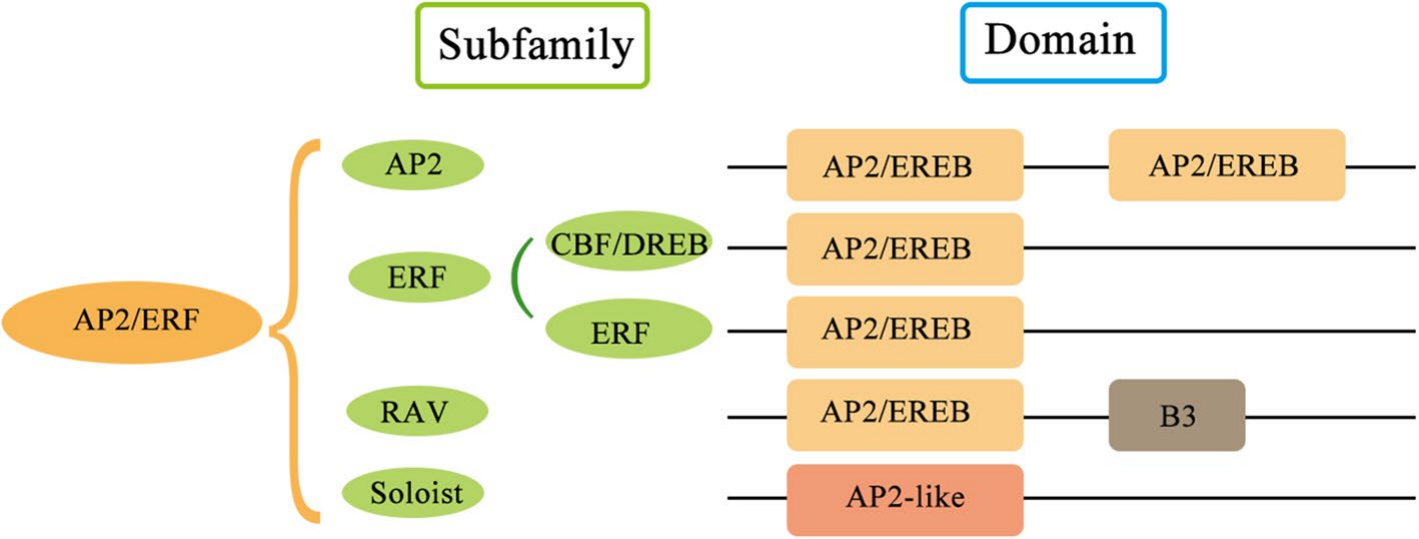
Schematic representation of domain structures of different types of AP2/ERFs. The position and amount of domains in different types of AP2/ERFs were illustrated. AP2, APETALA2; ERF, ethylene response factors; RAV, RELATED to ABSCISIC ACID INSENSITIVE 3/VIVIPAROUS 1; DREB, dehydration-responsive element-binding protein

The AP2/ERF superfamily can be catagorized into four families: AP2, ERF, RAV (Related to Abscisic acid insensitive3/Viviparous1), and Soloist, mainly based on the number of AP2/EREB domains and the biological function of the members (Feng, et al. 2020; Nakano, et al. 2006). Among them, the members of RAV and ERF family usually have a single AP2/EREB domain, while AP2 family members usually contain two (Fig. 1). For RAV family members, they usually contain a single AP2/EREB domain and another DNA-binding domain, B3 domain (Fig. 1). In addition, depending on conserved amino acid residues of the DNA binding domains, the ERF family members are further classified into two subfamilies, ERF and the CBF/DREB (C-repeat-binding factor/dehydration-responsive element-binding protein) (Fig. 1) (Lata and Prasad 2011; Nakano, et al. 2006; Sakuma, et al. 2002). Specifically, in CBF/DREB proteins, the 14th valine and the 19th glutamic acid are conserved in the DNA binding domain, while these two positions are occupied by alanine and aspartic in the DNA binding domain of ERF proteins, respectively (Liu, et al. 1998; Sakuma, et al. 2002). These two positions are located on the β-sheet in the DNA binding domain, which determines the DNA binding affinity and specificity of the two subfamilies, resulting in regulation of different sets of stress-responsive genes (Liu, et al. 1998; Sakuma, et al. 2002). For instance, members of CBF/DREB subfamily usually regulate ABA, drought, heat, and cold-responsive genes (Agarwal, et al. 2006), while members of the ERF subfamily preferentially regulate expression of genes associated with ethylene response, biotic stress, and disease resistance (Table 1) (Amorim, et al. 2017; Ohme-Takagi and Shinshi 1995).

As classical TFs, AP2/ERF proteins directly bind to the *cis*-acting elements within the promoter of targets, leading to activation or suppression of target genes. Different families of AP2/ERFs preferentially bind to different elements. For example, CBF/DREB subfamily members bind to DRE/CRE (Dehydration-Responsive or C-Repeat Element) motif with core sequence of A/GCCGAC on abiotic-stress responsive genes, while ERFs prefer to bind ERE (Ethylene-Response Element) motif with core sequence of AGCCGCC (named GCC-box) on the biotic-stress responsive genes (Xie, et al. 2019; Zhai, et al. 2022). Additionally, the B3 and AP2 domains of the RAV family members specifically bind to motifs with core sequence of CAACA and CACCTG, respectively (Kagaya, et al. 1999). However, some ERFs and CBF/DREBs in Arabidopsis bind both DRE/CRE and ERE elements, indicating they function in response to both biotic and abiotic stresses (Xie, et al. 2019). Additionally, multiple *cis*-elements have been reported to be recognized and bound by AP2/ERFs, such as HRPE (Hypoxia-Responsive Promoter Element), CE1 (Coupling Element 1), and others (Xie, et al. 2019). As aforementioned, the DNA-binding affinity of AP2/ERF proteins can be affected by certain amino acid residues of the AP2 domain, resulting in different DNA-binding specificity of different AP2/ERF proteins (Shoji, et al. 2013). Moreover, the binding affinity of AP2/ERFs can also be affected by flanking sequences of the *cis*-elements, leading to various binding specificity of AP2/ERFs to different target genes (Shoji, et al. 2013). The family specific preferential *cis*-element sequences were found to be conserved across a range of plant species including *Zea mays* (Liu, et al. 2013), *Oryza sativa* (Wan, et al. 2011), *Triticum aestivum* (Gao, et al. 2018), and *Glycine max* (Zhang, et al. 2009).

## AP2/ERFs play vital roles in MAPK cascades-mediated plant disease resistance

Mitogen-activated protein kinase (MAPK)-mediated signaling pathways are conserved among all eukaryotes. In plants, MAPKs usually act downstream of sensors/receptors that recognize endogenous stimuli (e.g. peptide ligands) or exogenous stimuli (e.g. PAMPs and environmental factors) to coordinate plant growth, development, and immunity (Zhang, et al. 2018; Zhang and Zhang 2022). Activated MAPKs mediate the phosphorylation of various downstream substrates, such as protein kinases, TFs, structural proteins, and other enzymes to activate cellular responses (Zhang, et al. 2018; Zhang and Zhang 2022).

**Fig. 2:**
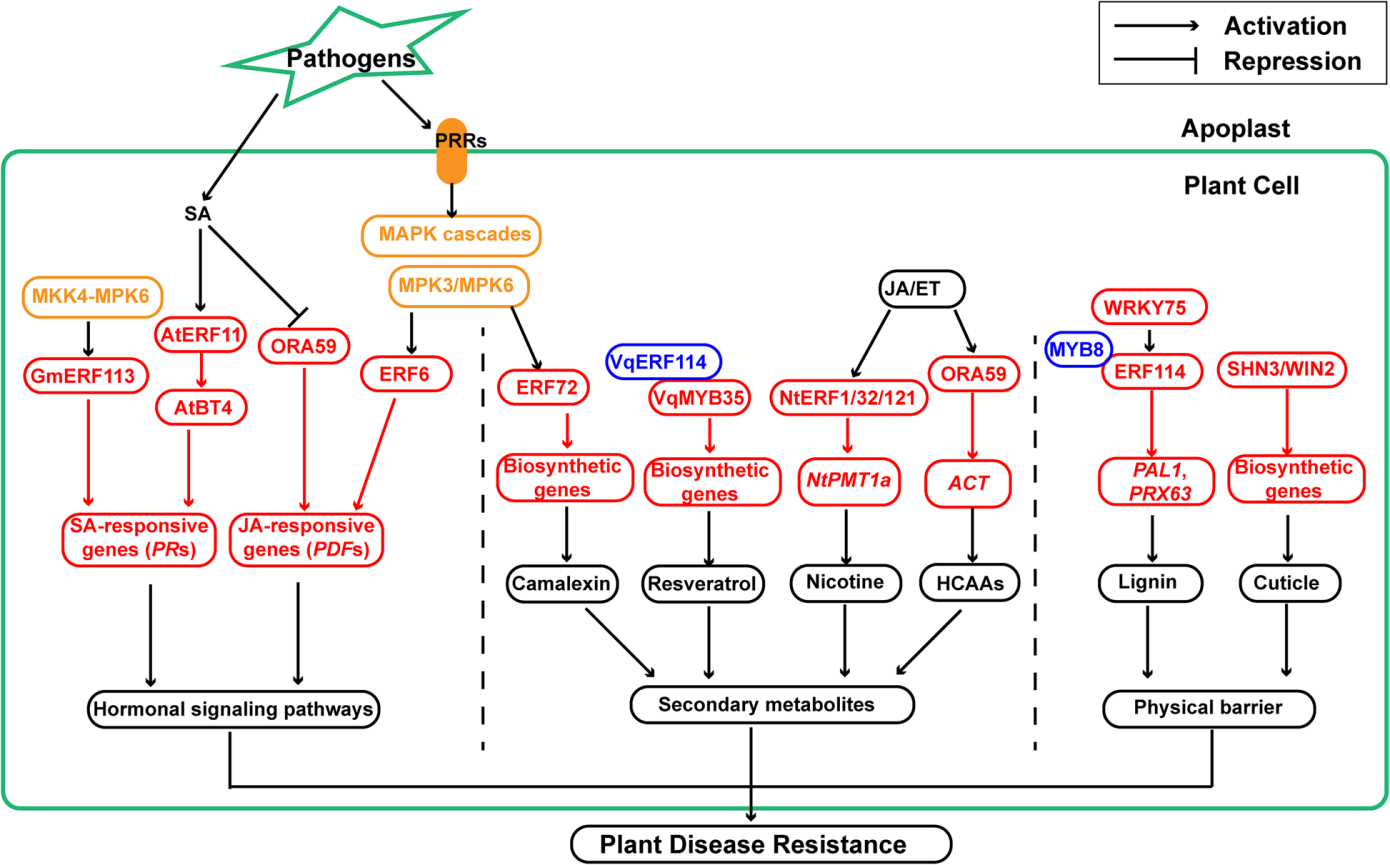
AP2/ERF TFs are involved in plant disease-resistance through multiple signaling pathways. In plant defence responses, pathogen-secreted molecules are perceived by membrane-localized PRRs, which subsequently induce cellular responses, such as MAPK signaling cascades. AP2/ERFs could serve as substrates of MAPKs (labeled in orange) and are involved in MAPK signaling-mediated plant defence responses. Moreover, AP2/ERFs serve as TFs to regulate genes involved in hormonal signaling pathways, secondary metabolites biosynthesis, and formation of physical barriers (cuticle and cell wall). AP2/ERFs are typical transcription factors, they usually modulate cellular responses at transcription level in the nucleus, as indicated by the red-labeled processes. Molecules labeled in blue indicate that they interact with and enhance the DNA-binding activity or transactivition activity of corresponding TFs

MAPK signaling pathways are among the early stage responses of PTI, and MAPK cascades have been widely demonstrated to be important signaling modules in plant disease resistance (Cheong, et al. 2003; Meng, et al. 2013; Zhang, et al. 2018; Zhang and Zhang 2022). To date, multiple AP2/ERF TFs have been determined to be substrates of MAPKs connecting plant defense responses (Table 1 and Fig. 2) (Bethke, et al. 2009; Cao, et al. 2018; Wang, et al. 2013). For example, a rice MAP kinase BWMK1 (blast and wounding-activated MAP kinase 1) phosphorylates and promotes binding affinity of OsEREBP1 (rice ethylene-responsive element-binding protein 1) to the GCC-box within the promoter of the *PR* genes, resulting in enhanced expression of these *PR*s and disease resistance (Cheong, et al. 2003). Additionally, two different groups reported that MPK3/MPK6, two partially redundant MAPKs in Arabidopsis, phosphorylate ERF6 and ERF72 to enhance disease resistance to *Botrytis cinerea* via different pathways (Li, et al. 2022a, b; Meng, et al. 2013). Specifically, MPK3/MPK6 phosphorylate ERF6 to increase its protein stability and DNA binding affinity to enhance the expression of defensive genes, including *PDF1.1* and *PDF1.2*, while MPK3/MPK6 phosphorylate ERF72 to promote its transactivation activity, leading to increased accumulation of camalexin and elevated immunity (Li, et al. 2022a, b; Meng, et al. 2013). Moreover, MAPK kinase 4 (GmMKK4)-GmMPK6 module phosphorylates GmERF113 to promote its protein stability and transcriptional activity, resulting in increased expression of defensive genes (e.g. *GmPR1* and *GmPR10-1*) and enhanced immune responses to *Phytophthora sojae* in *Glycine max* (Gao, et al. 2022).

## AP2/ERFs regulate plant disease resistance as transcriptional activators or repressors

As typical TFs, AP2/ERFs might act as transcriptional activator or repressor to regulate plant disease resistance by binding to corresponding targets. For instance, AP2/ERF transgenic plants develop immunity to a variety of pathogens by activating expression of multiple defense genes (Liu, et al. 2023; Zhu, et al. 2022). Specifically, overexpressing *GmERF113* in soybean (cultivar Dongnong 50) activates expression of *GmPR1* and *GmPR10-1* to enhance resistances to infection of *Phytophthora sojae* (Zhao, et al. 2017). Similarly, overexpressing *OsERF83* in rice significantly inhibits the lesion formation induced by fungal pathogen *Magnaporthe oryzae*, suggesting *OsERF83* plays a positive role in regulating resistance against rice blast (Tezuka, et al. 2019).

In some cases, AP2/ERFs act as transcriptional repressor to participate in plant disease resistance. For instance, AtERF9 is a transcription repressor binding to the GCC-box of *PATHGEN-INDUCIBE PLANT DEFENSIN* (*PDF1.2*) gene, and knocking out *AtERF9* significantly promotes *AtPDF1.2* expression, resulting in enhanced resistance to *Botrytis cinerea* (Maruyama, et al. 2013). Moreover, some AP2/ERF proteins contain a conserved C-terminal (L/F)DLN(L/F)xP motif, also known as EAR (ERF-associated amphiphilic repression) motif, and function to inhibit target gene expression (Fujimoto, et al. 2000). In yeast cells, EAR type StERF3 from tomato could bind to the promoter of *HIS3* gene via the GCC-box element (Tian, et al. 2015). In tomato, silencing *StERF3* promotes the expression of defensive genes (*PR1*, *NPR1*, and *WRKY1*), resulting in enhanced immunity to *Phytophthora infestans* (Tian, et al. 2015).

## AP2/ERFs integrates hormonal signaling to regulate plant disease resistance

SA, jasmonic acid (JA), and ET are the three major phytohormones that are extensively involved in plant defense responses. SA is mainly responsible for immune responses against biotrophic pathogens, while JA and ET signaling are synergistically regulated and are closely associated with immunity against necrotrophic pathogens and herbivores (Li, et al. 2019a, b; Peng, et al. 2021; Zhang, et al. 2023a, b, c). Moreover, SA and JA/ET usually regulate disease resistance in an antagonistic manner, but new evidence has proved that they may also function synergistically to regulate plant immunity against virus infection in rice (Li, et al. 2019a, b; Zhang, et al. 2023a, b, c).

SA is the most important defense hormone in plant immune system. SA regulates the immune responses either in local infection site or in systemic organs through the master regulator NPR1 in its signaling pathway (Peng, et al. 2021; Zhang and Li 2019). AP2/ERFs have been reported to regulate plant resistance by regulating SA biosynthesis or signaling pathway (Table 1 and Fig. 2) (Hawku, et al. 2021; Wang, et al. 2020). For instance, MdERF11 positively regulates defense responses against *Botryosphaeria dothidea* by promoting SA biosynthesis in apple (*Malus doemstica*) (Wang, et al. 2020). *ERF11* is induced upon treatment of SA and *Pst* DC3000, and disruption of *ERF11* suppresses SA-mediated defense responses, leading to decreased resistance to *Pst* DC3000 (Zheng, et al. 2019). Further analysis shows that ERF11 could activate the expression of *BT4* (BTB and TAZ domain protein 4), which encodes a positive regulator in SA-mediated immunity, by binding to its promoter through GCC-box (Zheng, et al. 2019). These data indicate that ERF11 acts upstream of BT4 to regulate SA response and disease resistance (Zheng, et al. 2019).

In addition to SA, AP2/ERFs are also involved in regulating disease resistance through JA/ET signaling pathway (Table 1 and Fig. 2). For instance, constitutive expression of *ERF5* or *ERF6* promotes expression of JA/ET-responsive genes, leading to enhanced immunity to *Botrytis cinerea*, a typical necrotrophic pathogen (Moffat, et al. 2012). Another AP2/ERF protein ORA59 is reported to promote JA/ET response by directly binding to the two GCC-boxes on the promoter of *PDF1.2* to elevate immune response to *Botrytis cinerea* (Huang, et al. 2022; Zarei, et al. 2011). In contrast to ORA59 trans-activating *PDF1.*2, AtERF9 is reported to repress *PDF1.2* expression by targeting to the GCC-box within its promoter, suggesting different AP2/ERFs might co-regulate the same target in either positive or negative manner (Maruyama, et al. 2013).

More importantly, AP2/ERFs are among key factors that mediate the crosstalk between signaling pathways of SA and JA/ET (Fig. 2). Analysis revealed that GCC-box motifs are present in the promoters of various JA/ET-responsive genes, and those motifs are critical for SA-induced repression of JA/ET-responsive genes (Van der Does, et al. 2013; Zander, et al. 2014). ORA59 plays key roles in JA/ET-mediated defense responses (Huang, et al. 2022; Zarei, et al. 2011), and SA could down-regulate both the transcription and protein accumulation of ORA59 (Van der Does, et al. 2013; Zander, et al. 2014). Thus, ORA59 stands at the middle of SA and JA/ET signaling to modulate the SA-mediated repression of JA/ET response. Moreover, ectopic expression of *MdERF100* promotes expression of *PR1* and *PDF1.2*, two marker genes of SA- and JA-signaling pathway, respectively, resulting in enhanced immune response to powdery mildew in Arabidopsis (Zhang, et al. 2021a, b, c).

In some cases, AP2/ERFs regulate disease resistance by modulating the biosynthesis of SA and JA simultaneously. For example, OsERF3 positively regulates resistance to herbivores by increasing the accumulation of SA, JA, and ET in rice (Lu, et al. 2011). In addition, SlERF2 also promotes accumulation of SA and JA to increase immune response to fungal pathogen *Stemphylium lycopersici* in tomato (Yang, et al. 2021a, b).

## AP2/ERFs regulate plant disease resistance by manipulating biosynthesis of secondary metabolites

Plant metabolites are traditionally categorized into two classes, primary and secondary. Primary metabolites are widely present in all plants and are necessary for plant growth and development, while secondary metabolites are involved in multiple physiological and biochemical processes, such as adaptation to harsh environments and defense responses (De Geyter, et al. 2012; Zhan, et al. 2022). Based on the core structures, secondary metabolites can be classified into three major groups: nitrogen/sulfur-containing compounds (e.g. alkaloids, glucosinolates, and cyanogenic glycosides), terpenoids/isoprenoids, and phenolics (Marone, et al. 2022). Secondary metabolites contribute to plant defencse responses via multiple pathways, including serving as antioxidants to scavenge ROS, being toxic to invaders (e.g. microbial pathogens, herbivores, and competing plant species), triggering the expression of defense-related genes, and accumulation of other metabolites (Zhan, et al. 2022).

TFs have been shown to be extensively associated with regulation of the secondary metabolites synthesis in response to biotic stresses, and AP2/ERFs are among them (Table 1 and Fig. 2) (Imano, et al. 2021; Nakayasu, et al. 2018; Shoji, et al. 2010). Resveratrol is a major stilbene-type phytoalexin characterized with antimicrobial activity in plants and medical properties in humans (Hart 1981; Jang et al. 1997). VqMYB35 directly binds to the promoters and activates the expression of stilbene synthase-encoding genes, and VqERF114 could interact with VqMYB35 and enhance its transactivation activity, leading to increased stilbene biosynthesis in *Vitis quinquangularis* (Wang and Wang 2019). Nicotine is a type of alkaloids that mainly produced in *Nicotiana* species, and it is involved in immune responses against insect and herbivore invasion (Zenkner, et al. 2019). JA and methyl JA (MeJA) promote nicotine formation by inducing the expression of putrescine N-methyltransferase-encoding gene (*NtPMT1a)*, which catalyzes the first committed step in nicotine pyrrolidine ring formation (Baldwin, et al. 1994; Sachan and Falcone 2002). A set of AP2/ERFs, including NtERF1, NtERF32, and NtERF121, activate the expression of *NtPMT1a* by binding specifically to its promoter, leading to elevated accumulation of nicotine and total alkaloid (Sears, et al. 2014). In addition to nicotine, JA/ET treatment also induced the biosynthesis of hydroxycinnamic acid amides (HCAAs), another type of antimicrobial metabolites that are associated with immune responses against necrotrophic pathogens (Campos, et al. 2014). Agmatine coumaryl transferease (ACT) is a key enzyme in HCAAs biosynthesis (Muroi, et al. 2009). Further investigations show that ORA59, a critical regulator in JA/ET-mediated defense signaling pathways, activates the *ACT* expression by binding to the GCC-box within its promoter, resulting in increased accumulation of HCAAs (Li, et al. 2018).

Additionally, AP2/ERFs have also been reported to regulate terpenoid indole alkaloids (TIAs) biosynthesis in both *Ophiorrhiza pumila* (Udomsom, et al. 2016) and *Catharanthus roseus* (Paul, et al. 2020, 2017), steroidal glycoalkaloids (SGAs) synthesis in both *Solanum lycopersicum* and *Solanum tuberosum* (Cárdenas, et al. 2016; Nakayasu, et al. 2018; Thagun, et al. 2016), and artemisinin production in *Artemisia annua* (Lu, et al. 2013; Yu, et al. 2012). These results confirm the critical roles of AP2/ERFs in modulating metabolism of secondary metabolites to regulate plant immunity.

## AP2/ERFs regulates plant disease resistance by mediating biosynthesis of physical barriers

In plant defense systems, plant cell wall is one of the key barriers that mirobes need to break to achieve successful colonization in host plant tissues. Plant cell wall functions in several facets to prevent pathogen invasion. First, cell wall is a physical barrier, as colonization inside host cells require breakdown of wall matrix by microbial-secreted cell wall-degrading enzymes (Miedes, et al. 2014). Upon cell wall breakdown, the cell wall-docked antimicrobial compounds are released and trigger downstream immune responses (Miedes, et al. 2014; Yang, et al. 2021a, b). Additionally, impaired cell wall integrity results in release of signaling molecules (e.g. damage-associate molecular patterns, DAMPs) and activation of specific immune responses, including SA and JA signaling pathways (Cantu, et al. 2008; Chowdhury, et al. 2019; Engelsdorf, et al. 2019).

Lignin, one of the key constituents of plant cell walls (CWs), is closely involved in determining the mechanical strength, antioxidation, and hydrophobicity of CWs (Cesarino 2019; Vanholme, et al. 2019). Upon pathogen invasion, the accumulated lignin offers a fundamental barrier to exclude invaders outside the host cells. It also limits the substance exchange between host and microbes, such as restricting mirobe-secreted toxins and enzymes entering into host cells, as well as the nutrients transportation from hosts to invaders (Dong and Lin 2021; Ma, et al. 2018). Thus, lignin has become a critical target for developing strategies to control plant diseases. Multiple investigations have reported the critical function of AP2/ERFs in regulating plant defense responses by manipulating lignin biosynthesis (Table 1 and Fig. 2). For example, overexpressing *GbERF1-like* significantly increases the transcripts of lignin biosynthetic genes, resulting increased lignin contents and enhanced resistance to *Verticillium dahliae* in upland cotton (*Gossypium hirsutum*) (Guo, et al. 2016). Two different groups report that ERF114 regulates disease resistance by manipulating lignin biosynthesis in both apple (*Malus domestica*) and Arabidopsis (Li, et al. 2022a, b; Liu, et al. 2023). Specifically, AtERF114 activates lignin binsynthetic gene *AtPAL1* expression by directly binding to its promoter, leading to increased accumulation of lignin and elevated immunity in Arabidopsis (Li, et al. 2022a, b). PEROXIDASEs (PRXs) catalyze the lignin formation through oxidative polymerization of three monolignol precursors (Marjamaa, et al. 2009). In apple, MdERF114 activates *MdPRX63* expression by directly binding to the GCC-box in its promoter, leading to increased lignin accumulation in apple roots and promoted immune responses against *Fusarium solani* (Liu, et al. 2023). Moreover, other TFs like MdWRKY75 and MdMYB8 promote the lignin formation by either regulating the expression or transcriptional activity of MdERF114, resulting in enhanced lignin deposition and disease resistance (Liu, et al. 2023).

In addition to the cell wall, the cuticle is another critical physical barrier that covers almost all the above ground organs of plants. The cuticle is composed of cutin and waxes, and plays critical roles in regulating epidermal permeability and nonstomatal water loss (Arya, et al. 2021; Sieber, et al. 2000). Similar with the function of cell wall, it not only defends against insects, fungi, and bacteria as physical barrier, but also acts as a chemical deterrent and activator of plant immune responses (Arya, et al. 2021). SHINE (SHN) transcription factors (TFs) belong to group V ERF subfamily and have been found to regulate the formation of the cuticle (Aharoni, et al. 2004; Riechmann and Meyerowitz 1998). Overexpressing *SlSHN3* in tomato promotes the accumulation of cutin monomers, resulting in a more permeable cuticle, which contributes to enhanced resistance to *Botrytis cinerea* and *Xanthomonas campestris* pv. *vesicatoria* (Bessire, et al. 2007; Buxdorf, et al. 2014). Moreover, application of cutin monomers extracted from *SlSHN3* overexpressing plants alters the expression of disease-related genes (e.g. *PR1a* and *AOS*), thereby restricting the development of disease symptom in wild-type plants (Buxdorf, et al. 2014). In addition, GhWIN2 (WAX INDUCER 2), a cotton (*Gossypium hirsutum*) homolog of SlSHN3, activates the cuticle biosynthetic genes and promotes cuticle formation in cotton (Li, et al. 2019a, b). However, silencing *GhWIN2* promotes resistance to *Verticillium dahliae*, probably because of the increased content of SA in cotton (Li, et al. 2019a, b), consistent with previous notions that AP2/ERFs integrate multiple pathways to regulate disease resistance in plants.

## Conclusions and future perspectives

The AP2/ERFs are critical stress-responsive TFs that are tightly associated with immune responses in plants. Here, we mainly summarize the functions of AP2/ERFs in plant defensive responses against microorganisms, such as fungi, bacteria, and viruses. Multiple AP2/ERF-encoding genes are induced by pathogens or defence-related phytohormones (e.g. SA, JA, or ET). Upon pathogen invasion, AP2/ERFs may serve as substrates of MAPKs to associate with MAPK signaling cascade-mediated defensive pathways (Table 1 and Fig. 2). Moreover, AP2/ERFs function as TFs to reprogram expression of defence-related genes involved in hormonal signaling pathways, secondary metabolism, and formation of physical barriers to regulate plant disease resistance (Tabel 1 and Fig. 2). Importantly, some AP2/ERFs may function as a signal hub or mediate the crosstalk between different signaling pathways. For example, while ORA59 serves as a master regulator in JA/ET-mediated defence response (Huang, et al. 2022; Zarei, et al. 2011), it can also be regulated by SA at both transcriptional and protein levels (Van der Does, et al. 2013; Zander, et al. 2014). Moreover, it is also directly involved in regulating biosynthesis of HCAAs, an important antimicrobial metabolite in plant defence system (Li, et al. 2018). Therefore, novel AP2/ERF family members that regulate plant disease resistance in multiple facets, as well as the underlying mechanism, need to be explored in the future researches.

At the current stage, with the rapid development of modern technologies, such as next generation sequencing, multi-omics analysis, the gene-editing system clustered regularly interspaced short palindromic repeats/Cas9 (CRISPR/Cas9), genome-wide association studies (GWAS), and others, the exploration and dissection of AP2/ERFs regulatory network will broaden our understanding of their roles in plant disease resistance. It is worth to mention that, in addition to transcriptional regulation, post-translational modification (PTM) of proteins are also tightly associated with the regulation of plant immunity (Gough and Sadanandom 2021; Withers and Dong 2017; Yin, et al. 2019). PTMs are highly specific alterations of protein structures, and most of them are rapid and reversible in plant cells (Jensen 2004). To date, more than 300 types of PTMs have been identified in plants, such as acetylation, ubiquitination, methylation, sumoylation, phosphorylation, glycosylation, and so on (Jensen 2004). PTMs usually affect the protein conformation, localization, stability, activity, and protein–protein interactions (Jensen 2004; Withers and Dong 2017). For AP2/ERFs, multiple reports have demonstrated their functions in plant immunity. For example, MAPKs target and phosphorylate AP2/ERFs to regulate their protein stability, DNA binding activity to regulate AP2/ERF-mediated plant disease resistance (Gao, et al. 2022; Li, et al. 2022a, b). In addition, a BTB/POZ domain protein GmBTB/POZ promotes the ubiquitination and degradation of AP2/ERF-like TF GmAP2, a negative regulator of plant resistance to *Phytophthora sojae*, resulting in enhanced resistance to *Phytophthora sojae* in soybean (*Glycine max*) (Zhang, et al. 2021a, b, c). Therefore, identification of PTMs of AP2/ERFs using multi-omics analysis will facilitate the dissection of molecular mechanisms underlying AP2/ERFs-regulated plant immunity. With the identification of novel AP2/ERFs, as well as the uncovering of underlying mechanism, AP2/ERFs can be potential candidates for disease resistance breeding in many crops.

## Data Availability

Not applicable.

## Abbreviations

ACT: Agmatine coumaryl transferease
AP2/ERF: APETALA2/ethylene response factor
CBF/DREB: C-repeat-binding factor dehydration-responsive element-binding protein
CE1: Coupling Element 1
CW: Cell wall
DRE/CRE: Dehydration-Responsive or C-Repeat Element
EAR: ERF-associated amphiphilic repression
EREB: Ethylene Responsive Element Binding Factor
ET: Ethylene
ETI: Effector-triggered immunity
HCAA: Hydroxycinnamic acid amide
HR: Hypersensitivity reaction
HRPE: Hypoxia-Responsive Promoter Element
MAPK: Mitogen-activated protein kinase
NLR: Nucleotide-binding domain, leucine-rich-repeat containing receptor
PAMP: Pathogen-associated molecular patterns
PDF1.2: PATHGEN-INDUCIBE PLANT DEFENSIN
PR: Pathogenesis-related
PRR: Pattern recognition receptor
PTI: Pathogen-associated molecular patterns-triggered immunity
ROS: Reactive oxygen species
SA: Salicylic acid
SGA: Steroidal glycoalkaloid
TIA: Terpenoid indole alkaloid
